# Annotating the Function of the Human Genome with Gene Ontology and Disease Ontology

**DOI:** 10.1155/2016/4130861

**Published:** 2016-08-22

**Authors:** Yang Hu, Wenyang Zhou, Jun Ren, Lixiang Dong, Yadong Wang, Shuilin Jin, Liang Cheng

**Affiliations:** ^1^School of Life Science and Technology, Harbin Institute of Technology, Harbin 150001, China; ^2^School of Software, Harbin Institute of Technology, Harbin 150001, China; ^3^School of Computer Science and Technology, Harbin Institute of Technology, Harbin 150001, China; ^4^Department of Mathematics, Harbin Institute of Technology, Harbin 150001, China; ^5^College of Bioinformatics Science and Technology, Harbin Medical University, Harbin 150081, China

## Abstract

Increasing evidences indicated that function annotation of human genome in molecular level and phenotype level is very important for systematic analysis of genes. In this study, we presented a framework named Gene2Function to annotate Gene Reference into Functions (GeneRIFs), in which each functional description of GeneRIFs could be annotated by a text mining tool Open Biomedical Annotator (OBA), and each Entrez gene could be mapped to Human Genome Organisation Gene Nomenclature Committee (HGNC) gene symbol. After annotating all the records about human genes of GeneRIFs, 288,869 associations between 13,148 mRNAs and 7,182 terms, 9,496 associations between 948 microRNAs and 533 terms, and 901 associations between 139 long noncoding RNAs (lncRNAs) and 297 terms were obtained as a comprehensive annotation resource of human genome. High consistency of term frequency of individual gene (Pearson correlation = 0.6401, *p* = 2.2*e* − 16) and gene frequency of individual term (Pearson correlation = 0.1298, *p* = 3.686*e* − 14) in GeneRIFs and GOA shows our annotation resource is very reliable.

## 1. Introduction

The human genome is the complete set of nucleic acid sequence for human beings [1]. Researches on sequence of the human genome aim at exploring the functions of genes [2–5]. Human genes consisting of sequences could play diverse roles based on their functions in molecular level in balancing the body. Once the balance is lost by lack or enhancement of the functions of genes, diseases could be induced [6–9].

Previous studies focused on identifying the functions of the protein-coding genes in molecular level based on their encoded proteins. For example, through investigating p53 protein, Brain and Jenkins [10] exposed that TP53 gene is potentially capable of inhibiting mammalian replicative DNA synthesis by blocking the DNA strand separation step during replication origin recruitment. Based on a case control study, Benzon Larsen et al. [11] determined that ADH polymorphisms, which modify the rate of ethanol oxidation to acetaldehyde, were associated with breast cancer risk.

As a growing number of protein-coding genes identified, lots of functional terms emerged. For ease of comparing the functions of genes, these terms needed to be normalized. To this end, ontology was introduced to standardize the functional terms of genes. Among existing ontologies, Gene Ontology (GO) [12] is one of the earliest and most frequently used vocabularies, which focuses on describing biological process (BP), molecular function (MF), and cell component (CC) of genes. Since appearing in 2000, a large number of databases recording the functions of genes were annotated to the GO. The functional annotation of human protein-coding genes was provided at GO Annotation (GOA) databases [13], which involves a nonredundant set of annotations to the human proteome. In comparison with the GO, Disease Ontology (DO) [14] focuses on standardizing the functional terms of genes at phenotype level. And disease terms in Gene Reference into Function (GeneRIF) [15] were annotated to the DO [16–18].

Recently, large-scale sequence analysis at genomic and transcriptomic level has shown that more than 98% of genome sequence cannot encode protein [19, 20], and microRNA genes and long noncoding RNA (lncRNA) genes constitute a large portion of them [21]. In comparison with protein-coding genes, the functions of microRNA genes and lncRNA genes are difficult to be identified [22]. However, these noncoding genes play an important role at molecular level and phenotype level [23–27]. For example, at molecular level, qPCR and in silico hybridization revealed that miR-124 and miR-155 can be directly involved in the transcriptional regulation of Runt-related transcription factor 2 (RUNX2) and receptor activator of nuclear factor kappa-B ligand (RANKL) genes [28]. At phenotype level, Huang et al. identified that underexpression of miR-345 is associated with prostate cancer [29]. At present, microRNA- and lncRNA-related diseases in HMDD [30] and LncRNADisease [31] have been manually annotated by Medical Subject Headings (MeSH) [32]. And several recent works proved more relationship between miRNA and diseases would be detected yet [33–35].

Although a few of databases have been annotated to gene functional vocabularies, a comprehensive annotation resource recording the functions of human genes had not yet appeared. For example, in our knowledge, no databases of noncoding genes were annotated to functional vocabularies at molecular level. This may be caused by the lack of resources that record the functions of protein-coding genes and noncoding genes simultaneously. Fortunately, GeneRIFs [15] provides a brief (up to 255 character) functional description of each gene in the NCBI database, and these functional descriptions could be annotated to vocabularies, such as DO and GO.

In this paper, we presented a framework, Gene2Function, to annotate the function of human genome with GO and DO. After annotating GeneRIF, a comprehensive resource involving protein-coding genes, microRNA genes, and lncRNA genes could be obtained. The resource could be accessed from http://www.bio-annotation.cn/gene2function/.

## 2. Results

### 2.1. Mapping Genes to Gene Ontology and Disease Ontology

After annotating GeneRIFs by GO and DO (see Section 3), 288,869 associations between 13,148 mRNAs and 7,182 terms, 9,496 associations between 948 microRNAs and 533 terms, and 901 associations between 139 lncRNAs and 297 terms were obtained. The statistical information is shown in Table 1.

**Figure 1:**
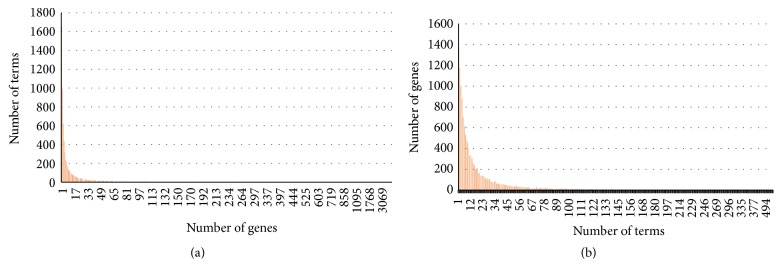
Distribution of functional terms and genes in the annotation results. (a) Histogram of the number of genes associated with individual functional term. (b) Histogram of the number of functional terms associated with individual gene.

Figure 1(a) demonstrates the histogram of the number of genes associated with terms of GO and DO in the annotation results. 1,657 functional terms (23.0%) are associated with only one gene, while 3,924 functional terms (54.5%) are associated with more than three genes. The histogram of the number of terms associated with individual gene is shown in Figure 1(b). 1,375 genes (9.9%) are associated with only one functional terms, while 10,273 genes (74.3%) are associated with more than three genes.

The top ten terms ordered by the number of gene annotations and the top ten genes ordered by the number of term annotations are shown in Tables 2 and 3, respectively. Not surprisingly, several general terms in the top layer of the DAG have a larger number of genes associated with them, such as cell, binding, and developmental process (Table 2). The most prevalent disease terms appearing in the annotation result is cancer, which is associated with 3,139 genes (22.7% of all the terms). When we look at the genes associated with many terms, TP53 is the most prevalent genes appearing in the annotation result, which is associated with 828 terms (11.5% of all the genes).

### 2.2. Comparing with Existing Ontology Annotation Resources

To validate the performance of our annotation result, we compared the result with the previous prevalent annotation resources GOA [13], in which human gene is manually annotated to GO. To ensure the exact evaluation, DO annotations of GeneRIFs were discarded, and annotations Inferred from Electronic Annotations (IEA) of GOA were removed.

In total, we obtained 196,423 associations between 4,613 GO terms and 13,107 genes in GeneRIFs and 168,246 associations between 13,920 GO terms and 16,724 genes in GOA. Only 10,658 associations and 3,375 GO terms appeared in both annotation resources. In comparison, both of them have more common genes (11,816).

**Figure 2:**
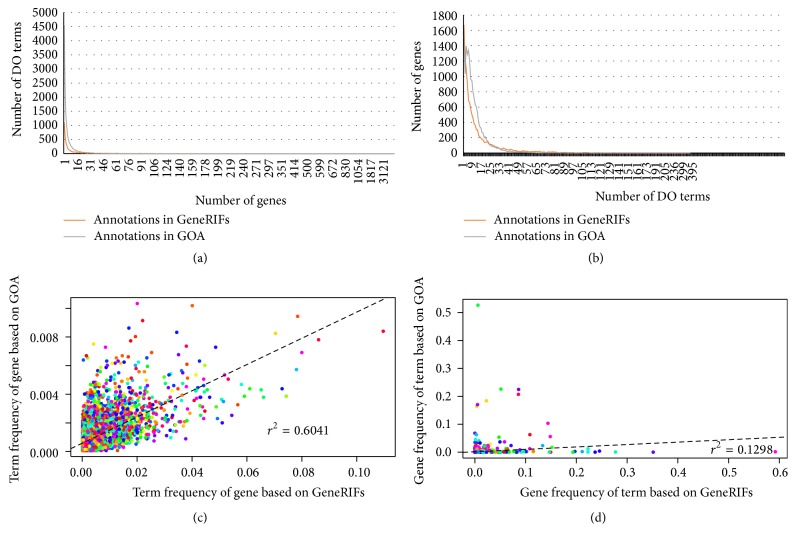
The comparison of annotations in GeneRIFs and with annotations in GOA. (a) Histogram of the number of genes associated with individual GO term. (b) Histogram of the number of DO terms associated with individual gene. (c) The correlation between term frequency of gene by GeneRIFs and GOA. (d) The correlation between gene frequency of term by GeneRIFs and GOA.

Figures 2(a) and 2(b) demonstrate the histogram of the number of genes per GO term, and the histogram of the number of GO terms per gene in annotations of GeneRIFs and GOA, respectively. Obviously, more GO terms (4,545) could be annotated to only one gene in GOA than that (1,114) in GeneRIFs. In contrast, more genes (1,671) could be annotated to only one term in GeneRIFs than that (1,499) in GOA.

In order to evaluate the consistency, we compared the term frequency of individual gene and gene frequency of individual term in GeneRIFs and GOA. As a result, term frequency of individual gene in GeneRIF was significant positively correlated with it in GOA (Pearson correlation *γ* ^2^ = 0.6401, *p* = 2.2*e* − 16; Figure 2(c)), and gene frequency of individual term in GeneRIF was also significantly positively correlated with it in GOA (Pearson correlation *γ* ^2^ = 0.1298, *p* = 3.686*e* − 14; Figure 2(d)). Considering that GOA is most frequency used annotation resource, annotations of GeneRIFs should be also reliable.

### 2.3. A Network Visualization Based on the Functional Annotation of the Human Genome

**Figure 3:**
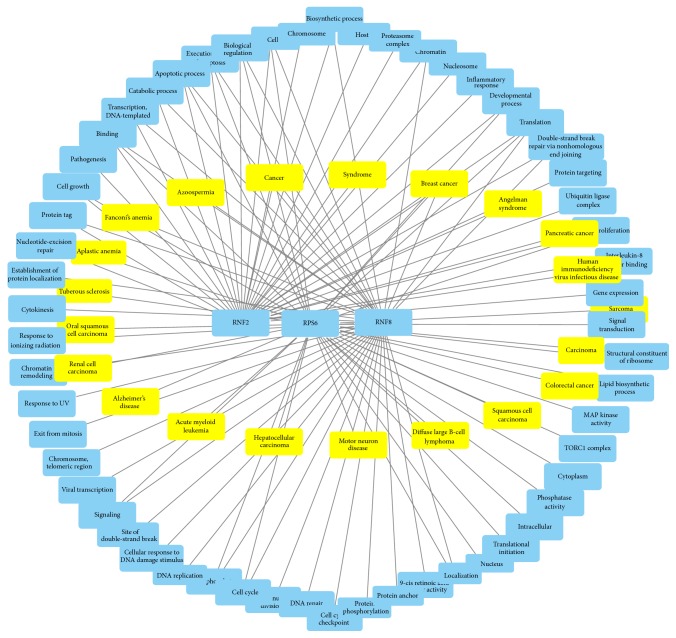
A bipartite network demonstrating the relationship between genes and terms. Rectangles with yellow represent DO terms, three rectangles with blue in the center of the figure indicate DO terms, and other rectangles are GO terms. An edge is placed between a gene and a term of GO and DO if the gene relates with the term.

Information in the annotation result can be used to describe the relationship among multiple genes or multiple terms. To this end, we create a bipartite network that describes the relationships between three genes (RNF2, RNF8, and RPS6) and 79 terms (Figure 3). Within this network, 33 terms are annotated to RNF2, 37 terms are annotated to RNF8, and 37 terms are annotated to RPS6. At the centre of the figure, 6 terms involving translation, execution phase of apoptosis, breast cancer, biological regulation, binding, and apoptotic process are related to all of these three genes. Using our annotation result, one can create this type of bipartite network as needed.

## 3. Materials and Methods

### 3.1. Data Collection

#### 3.1.1. GeneRIF

GeneRIF was downloaded in June 2016 (Table 4). It involves five columns for describing tax identifier, NCBI gene ID, PubMed Unique Identifier (PMID), updated date, and function description. After extracting function descriptions of human genes, 650,079 descriptions remained.

#### 3.1.2. Normalized Gene Symbol Vocabulary

The Human Genome Organisation Gene Nomenclature Committee (HGNC) [36] is responsible for approving unique symbols and names for human loci, including protein-coding genes and noncoding genes, to allow unambiguous scientific communication. In this paper, genes in GeneRIFs were normalized to HGNC gene symbols.

#### 3.1.3. Ontologies and Annotations

**Figure 4:**
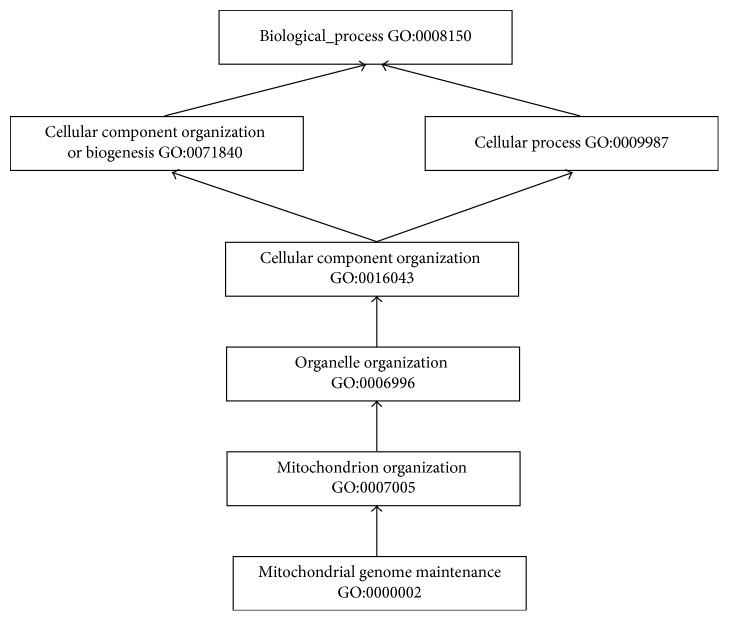
A subgraph of the DAG for BP term “Mitochondrial genome maintenance (GO:0000002).” The arrow symbol represents an “IS_A” link of GO. For example, “Mitochondrial genome maintenance (GO:0000002)” is linked to “Mitochondrion organization (GO:0007005)” by an “IS_A” relationship.

As shown in Figure 4, GO organized BP terms in the Directed Acyclic Graph (DAG) by “IS_A” relationship. Currently, GO contains 55,565 “IS_A” relationships between 28,654 BP terms, 12,375 “IS_A” relationships between 10,159 MF terms, and 5,618 “IS_A” relationships between 3,907 CC terms. GOA was compared with our annotation result. After removing IEA and getting rid of duplicate records of GOA, 168,246 associations between 13,920 GO terms and 16,724 genes remained.

DO is a first ontology to organize terms around human disease, which describes each disease by a unique identifier, a disease name, and its synonymous. In the current version, it involved 7,124 “IS_A” relationships between 6,920 disease terms.

### 3.2. Method for Annotating Human Genome

**Figure 5:**
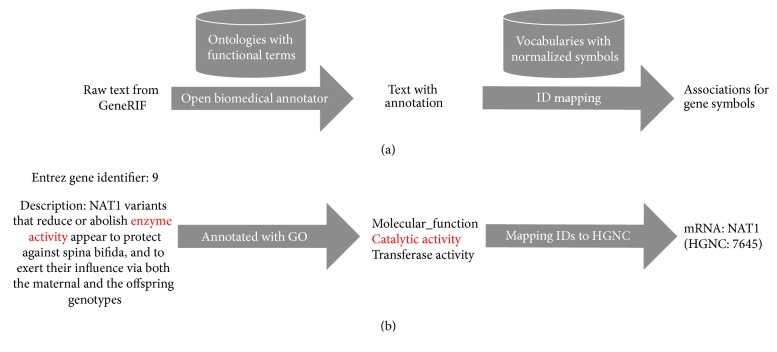
Diagram of functional annotation of human genome. (a) A framework to annotate functional description of human genome to ontologies. (b) An example of annotating a GeneRIF.

As shown in Figure 5(a), we presented a framework, Gene2Function, to annotate the function of human genome. Firstly, a raw text of GeneRIF with functional description should be annotated by a text mining tool named Open Biomedical Annotator (OBA) [37], which provided an ontology-based web service that annotates public datasets with biomedical ontology concepts based on their textual metadata. As a result, the functional description will be mapped to the corresponding ontologies, such as GO and DO. Then, the Entrez gene identifier will be converted into a normalized gene symbol. Here, HGNC was exploited for normalizing and labelling the locus type of gene, such as protein-coding genes, microRNA genes, and lncRNA genes. Finally, each GeneRIF could be annotated to a triple involving gene symbol, locus type, and functional description.

All the GeneRIFs could be annotated based on the annotation framework.  Figure 5(b) gives an example of annotating a GeneRIF with GO. “Enzyme activity” is a synonym of “catalytic activity (GO:0003824),” which was identified by OBA. And Entrez gene identifier “9” was converted into “NAT1 (HGNC:7645)” based on HGNC. Through the annotation framework, the annotation triple “mRNA, NAT1, catalytic activity” could be obtained.

## 4. Discussion

The importance of the functional annotations of genes had been reflected in the previous annotation resource, such as GOA. Unfortunately, functional annotation resources of noncoding RNA are very few, which lead to the lack of a comprehensive annotation resource involving protein-coding genes, microRNA genes, and lncRNA genes. With the largest number of noncoding genes in the human genome, it is urgent to provide functional annotation of these genes. In this study, we presented a framework, Gene2Function, for annotating GeneRIFs. As a result, a comprehensive functional annotation resource of human genome was obtained based on the framework, which could be accessed at http://www.bio-annotation.cn/gene2function/. To evaluate the reliability, our annotation result was compared with a prevalent resource GOA. Subsequently, a network visualization of connectivity of genes by their functional terms shows the usability of the annotation result.

The annotation framework is based on a text mining tool OBA [37]. Under the framework, the functional terms of descriptions of GeneRIFs were annotated to GO and DO terms. And gene symbols were mapped to a normalized vocabulary of human gene HGNC [36], which makes it easy to distinguish the locus type of gene, such as protein-coding RNA, microRNA, and lncRNA.

The consistency test of the GeneRIFs and GOA (Figures 2(c) and 2(d)) shows the reliability of our annotation result. Because of a small amount of common associations between genes and GO terms in both annotation resources, they could be complementary in the usage of protein-coding RNA annotation. More GO terms were annotated in GOA (see Section 2) suggesting it is more deep and serious than our annotation results. In comparison, advantage of GeneRIFs is that not only protein-coding genes but also microRNA genes and lncRNA genes could be annotated with GO and other function terms (Table 1).

## Figures and Tables

**Table 1:** The statistical information of associations between genes and terms.

The number of genes	The number of terms	The number of associations between genes and terms
mRNA		
13,148	7,182	288,869
MicroRNA		
948	533	9,496
lncRNA		
139	297	901

**Table 2:** The top ten terms ordered by the number of gene annotations.

Term ID	Term name	Number of genes
GO:0005623	Cell	7,524
GO:0005488	Binding	5,011
GO:0065007	Biological regulation	4,846
GO:0023052	Signaling	4,466
GO:0032502	Developmental process	3,521
GO:0009058	Biosynthetic process	3,346
DOID:162	Cancer	3,139
GO:0006351	Transcription, DNA-templated	3,121
DOID:305	Carcinoma	3,069
GO:0040007	Growth	3,011

**Table 3:** The top ten genes ordered by the number of term annotations.

HGNC gene ID	Gene symbol	Number of functional terms
HGNC:11998	TP53	828
HGNC:11892	TNF	792
HGNC:6018	IL6	683
HGNC:12680	VEGFA	669
HGNC:11766	TGFB1	664
HGNC:3236	EGFR	560
HGNC:7176	MMP9	521
HGNC:391	AKT1	517
HGNC:7794	NFKB1	494
HGNC:6025	CXCL8	473

**Table 4:** Data sources.

Data source	Web site (date of download)
GeneRIF	http://www.ncbi.nlm.nih.gov/gene/about-generif (Jun 2016)
HGNC	http://www.genenames.org/ (Jun 2016)
GO & GOA	http://geneontology.org/ (Jun 2016)
DO	http://disease-ontology.org/ (Jun 2016)
